# Symbiont polyphyly, co-evolution, and necessity in pentatomid stinkbugs from Costa Rica

**DOI:** 10.3389/fmicb.2014.00349

**Published:** 2014-07-15

**Authors:** Kalia S. I. Bistolas, Reid I. Sakamoto, José A. M. Fernandes, Shana K. Goffredi

**Affiliations:** ^1^Biology Department, Occidental CollegeLos Angeles, CA, USA; ^2^Instituto de Ciências Biológicas, Universidade Federal do ParáBelém-Pará, Brazil

**Keywords:** symbiosis, stinkbug, pentatomid, phytophagous, neotropical, *Erwinia*

## Abstract

Interdomain symbioses with bacteria allow insects to take advantage of underutilized niches and provide the foundation for their evolutionary success in neotropical ecosystems. The gut microbiota of 13 micro-allopatric tropical pentatomid species, from a Costa Rican lowland rainforest, was characterized and compared with insect and host plant phylogenies. Like other families within the Pentatomomorpha, these insects (within seven genera—*Antiteuchus, Arvelius, Edessa, Euschistus, Loxa, Mormidea*, and *Sibaria*) house near-monocultures of gamma-proteobacteria in midgut crypts, comprising three distinct lineages within the family Enterobacteriaceae. Identity of the dominant bacteria (78–100% of the recovered 16S rRNA genes) was partially congruent with insect phylogeny, at the level of subfamily and tribe, with bacteria closely related to *Erwinia* observed in six species of the subfamily Pentatominae, and bacteria in a novel clade of Enterobacteriaceae for seven species within the subfamilies Edessinae and Discocephalinae. Symbiont replacement (i.e., bacterial “contamination” from the environment) may occur during maternal transmission by smearing of bacteria onto the egg surfaces during oviposition. This transmission strategy was experimentally confirmed for *Sibaria englemani*, and suspected for four species from two subfamilies, based on observation of egg probing by nymphs. Symbiont-deprived *S. englemani*, acquired via egg surface sterilization, exhibited significantly extended second instars (9.1 days compared with 7.9 days for symbiotic nymphs; *p* = 0.0001, Wilcoxon's rank with Bonferroni correction), slower linearized growth rates (*p* = 0.005, Welch 2-sample *t*-test), and qualitative differences in ceca morphology, including increased translucency of crypts, elongation of extracellular cavities, and distribution of symbionts, compared to symbiotic nymphs. Combined, these results suggest a role of the symbiont in host development, the reliable transference of symbionts via egg surfaces, and a suggestion of co-evolution between symbiont and tropical pentatomid host insects.

## Introduction

Phytophagous insects represent one of the most diverse groups of metazoans, comprising a significant proportion of animal biodiversity in terrestrial biomes (Gilbert and Smiley, 1978). Due to their range of nutritional strategies, life histories, and ecological dominance in terrestrial habitats, herbivorous insects have emerged as ideal models for the study of symbioses. Nearly all of these insects associate with a complement of microbial symbionts, underpinning their evolutionary success by mediating relationships with respective host plants (Douglas, 2013; Joy, 2013). For example, bacterial symbionts are known to play a combination of roles to promote host fitness, including: (1) host plant allelochemical remediation, exemplified by conversion of 1-naphthyl acetate and tannic acid to metabolically-benign aromatic alcohol derivatives by symbionts of the columbine aphid (*Kakima essigi*) and maize weevil (*Sitophilus zeamais*; Shen and Dowd, 1991), (2) nutritional provisioning, illustrated by leucine and tryptophan synthesis by *Buchnera aphidicola* housed in bacteriocytes in aphids (*Acyrthosiphon* spp.; Douglas, 1998; Moran et al., 2003), and (3) mediation of host signaling, such as *Burkholderia* symbiont-regulated gene expression patterns of cysteine-rich secretion proteins in midgut tissues in the bean bug (*Riptortus pedestris;* Futahashi et al., 2013). These symbioses illustrate microbial governance of insect ecology and the role of bacteria in catalyzing resource-driven sympatric speciation by facilitating specialization to novel host plants (Klepzig, 2009; Joy, 2013).

Stinkbugs, in general, have been the subject of numerous studies, and encompass several families within the Heteroptera, including Acanthostomidae, Coreidae, Plataspidae, and Pentatomidae. These insects are important contributors to overall herbivory in all major biogeographic provinces and are well-known agriculture pests, causing damage to crops by piercing plant tissues (Panizzi, 1997; Corrêa-Ferreira and de Azevedo, 2002; Golden et al., 2006; Hosokawa et al., 2007). They impact host plant primary productivity and fecundity positively through pollination and antagonistic interactions with other host plant herbivores and negatively through selective herbivory and tissue damage, including insertion and laceration via stylet-sheaths during phloem feeding, and crushing of mesophyll cells during fluid extraction (Greig, 1993; Hori, 2000; Silva and Oliveria, 2010; Whitehead and Bowers, 2014). In particular, the cosmopolitan Pentatomidae is comprised of more than 4100 species worldwide, but is most diverse in the tropics (Schuh and Slater, 1995). This family is divided into eight subfamilies, three of which are represented in this study: Pentatominae, Edessinae and Discocephalinae. Pentatominae is the largest subfamily with more than 2800 species, while Edessinae and Discocephalinae, both of which are only found in neotropical regions, each comprise ~300 species (Schuh and Slater, 1995). The breadth of neotropical pentatomid diversity lends itself to the exploration of a spectrum of symbiotic partnerships, and the role that these partnerships play in structuring globally important ecosystems.

For at least nine primarily seed- and phloem-feeding pentatomid species, and some close relatives, bacteria have been observed in specialized posterior midgut structures, or ceca, and are thought to facilitate the utilization of a sap-based diet by compensating for deficient nutrients, including both amino acids and vitamins (Abe et al., 1995; Fukatsu and Hosokawa, 2002; Prado et al., 2006; Prado and Almeida, 2009b; Prado et al., 2009; Hosokawa et al., 2010; Nikoh et al., 2011; Zucchi et al., 2012). Symbiont elimination studies in temperate pentatomids illustrate the potential utility of these microbes. Individuals whose symbionts were eliminated exhibited increased mortality, longer durations between instars, delayed adult emergence, and reduced fecundity, indicative of a probable, though controversial, beneficial relationship between the bacteria and host (Hosokawa et al., 2007; Prado and Almeida, 2009b; Prado et al., 2010; Kikuchi et al., 2012; Taylor et al., 2014). This illustrates the potential of resident gut microbes to alter the ontogeny, morphology, and physiology of their stinkbug hosts, impacting their fitness, biogeography, evolution, and ability to support tropical food web architecture (Ferrari and Vavre, 2011; Su et al., 2013).

**Figure 1:**
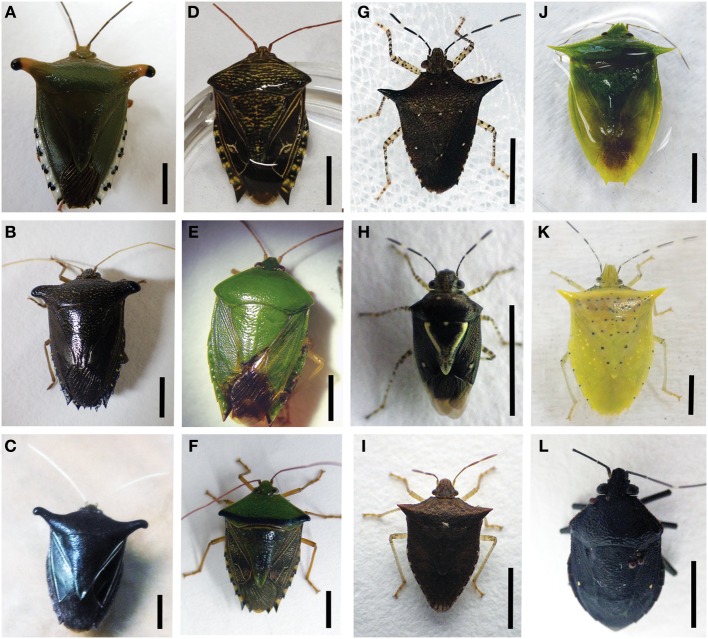
**Pentatomid specimens used in this study. (A)** *Edessa* n sp 1 **(B)** *Edessa bugabensis* **(C)** *Edessa jugata* **(D)** *Edessa* aff. *irrorata* **(E)** *Edessa junix* **(F)** *Edessa eburatula* **(G)** *Sibaria englemani* **(H)** *Mormidea* aff. *ypsilon* **(I)** *Euschistus* sp. **(J)** *Loxa* sp. **(K)** *Arvelius porrectispinus* **(L)** *Antiteuchus costaricensis*. *Mormidea collaris*, not shown. All scale bars, 5 mm.

It has been suggested recently that bacterial status (i.e., presence, diversity, and pervasiveness) is a necessary and integral component of investigations of insects, especially those less studied in the tropics, and that we will only truly understand their nutritional ecology in light of this important piece of the puzzle (Douglas, 2009, 2013; Hirose et al., 2012). Prado and colleagues revealed high symbiont-host insect specificity, particularly among recently diverged taxa, suggestive of co-speciation (Prado et al., 2006; Prado and Almeida, 2009a). However, a larger scope of pentatomid taxa must be evaluated. In this study, we provide evidence for an association between 13 species of Costa Rican pentatomid, within seven genera (Figure 1), and single dominant microbes within three lineages of the Enterobacteriales order of the Gammaproteobacteria. While research on other members of the Heteroptera suggest that microbial symbionts have influenced the evolutionary success of these insects, very little is known about the presence and persistence of bacteria in the digestive systems of tropical pentatomids.

## Methods

### Specimen collection

Insects were collected in northern Costa Rica at La Selva Biological Station (Organization for Tropical Studies), a 1500-hectare ecological reserve with a wet neotropical lowland climate (McDade et al., 1994). The station, located at the confluence of the Sarapiqui and Puerto Viejo rivers in the province of Heredia, Costa Rica (10°26′N, 83°59′W), covers ~6 sq miles, and is home to 30 pentatomid species (out of 170 species known to Costa Rica, James Lewis, *Pers. Commun.*). Collection and export permits were acquired through the Organization for Tropical Studies and the Costa Rican Ministry of the Environment and Energy (079-2013-SINAC). All pentatomids examined in this study (Figure 1) were collected using nets and plastic containers, within a ~1 km^2^ area near the central laboratory, with the exception of *Loxa, Euschistus*, and some *Mormidea* specimens, which were collected ~5 km away. All were either preserved immediately for molecular and microscopic analysis, or kept alive for natural history observations, captive breeding, and rearing of nymphs. Specimens were routinely observed and collected on specific host plants, including *Piper sancti-felicis* for *Sibaria englemani*, legumes for *Loxa* sp., grasses within the Poaceae for *Mormidea* and *Euschistus* species, *Handroanthus chrysanthus*, and *Pentaclethra macroloba* for *Edessa* n. sp 1, and *Guatteria amplifolia* for *Edessa* n. sp 3. *Antiteuchus costaricensis, Arvelius porrectispinus*, the various other *Edessa* specimens were found in association with man-made structures such as UV light traps, window screens, and wooden walls. Most specimens were examined using a Leica S8APO stereomicroscope and photographed with a Nikon Coolpix P6000 digital camera. Identities of the host insects were determined by morphology (Fernandes, personal observation).

### Molecular analyses

**Figure 2:**
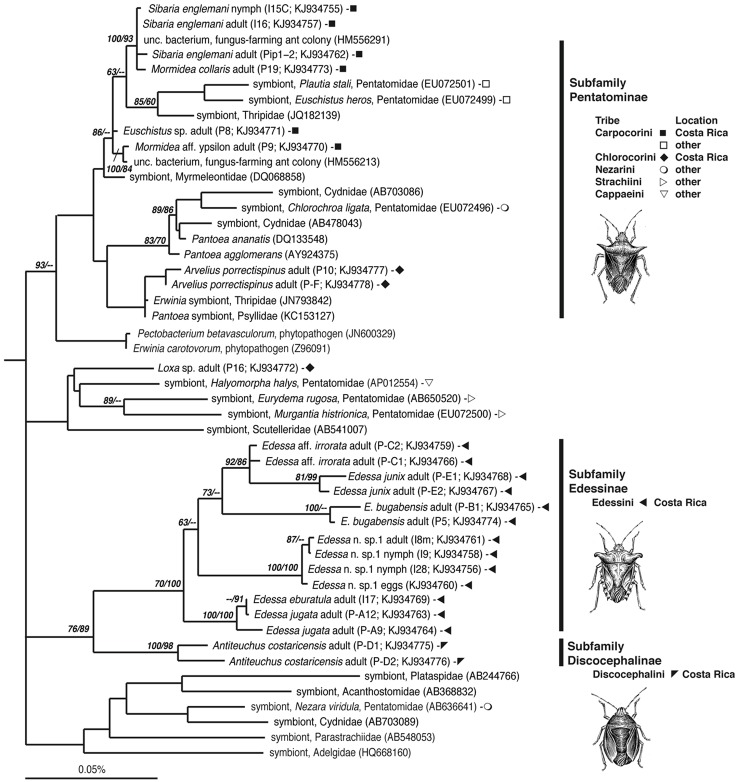
**Phylogenetic relationships of gammaproteobacteria associated with Pentatomids examined in this study, based on sequence divergence within the 16S rRNA gene**. Additional sequences were obtained from GenBank and compiled and aligned with our 16S rRNA sequences from Costa Rican insects (shown as filled symbols), using the ARB automated alignment tool with subsequent manual refinements. For near full-length representatives and closest relatives, maximum parsimony (MP) analysis was conducted with *Aeromonas veronii* (X71120) as an outgroup, not shown. Numbers next to nodes correspond to bootstrap values >60, based on 5000 neighbor-joining and 200 parsimony replicates, respectively.

Adult specimens preserved immediately in 70% ethanol were dissected and DNA was extracted from the ceca of each individual using the Qiagen DNeasy kit, according to the manufacturers instructions (Qiagen, Valencia, CA). Whole specimens for 1st instar nymphs, and total abdominal tissue for 2nd–5th instar nymphs preserved in 70% ethanol were also extracted as described above. To assess whether insects hosted internal bacteria, a 1465-bp fragment of the bacterial 16S rRNA gene was PCR-amplified using bacteria-specific primers (27F and 1492R; Lane, 1991). Clone libraries of bacterial 16S rRNA amplicons were constructed from each insect, using the TOPO TA cloning kit (Invitrogen, Carlsbad, CA). Transformants were grown overnight in LB broth with 50 μg ml^−1^ kanamycin, and screened directly for the presence of inserts using M13F and M13R vector primers and the thermal cycling conditions of an 8 min initial denaturation, followed by 30 s each of denaturation at 94°C, annealing at 54°C, elongation at 72°C (30 cycles), and a final 6 min of elongation at 72°C. In all cases, M13 amplicons were digested first with *HaeIII* (according to manufacturers instructions; New England Biolabs) in order to observe diversity and select unique samples for sequencing. In total, 19–48 clones were sequenced for each library, and revealed 78–100% dominance by a single bacterial OTU (97% similarity; Table 1). To selectively amplify the dominant bacteria within the *Sibaria* (Carpocarini) bacterial clade and the *Edessa* bacterial clade (Figure 2), diagnostic symbiont-specific primers were designed *in silico* (Sib_1F; 5′- GAAACTGCCC-GATGGAGG-3′ and Ed_1F; 5′- GGATCTACCTAGTGGAGGG-3′, respectively), and paired with 1492R to amplify a 16S rRNA product length of 1412-bp. This allowed for rapid screening of the presence of the dominant bacteria in eggs, nymphs, and field collected adults. Thermal cycling conditions included 60 s each of denaturation at 94°C, annealing at 54 and 52°C, for general and symbiont-specific 16SrRNA, respectively, elongation at 72°C (25 cycles), and a final extension at 72°C for 6 min. M13 amplicons, or 16S rRNA gene products sequenced directly, were cleaned prior to sequencing with MultiScreen HTS plates (Millipore Corporation, Bedford, MA). Samples were sequenced via ABI sequencing technology (Laragen, Inc., Culver City, CA). Sequences were assembled, edited, and aligned using Sequencher v4.10.1 (GeneCodes Corp.).

**Table 1:** **Summary of bacterial 16S rRNA clone library results for all individuals**.

**Subfamily**	**% Erwinia**	**% Enterobacteraceae^a^**	**% Unk/other**	**# of clones**
**PENTATOMINAE**				
*Sibaria englemanni*				
Adult	84	–	16	38
Adult	90	–	10	48
Adult	98	–	2	46
Adult	88	–	12	24
Nymph (5th instar)	92	–	8	13
Nymph (5th instar)	98	–	2	45
Nymph (5th instar)	98	–	2	44
Nymph (1st instar)	98	–	2^b^	45
Egg	94	–	6^c^	31
*Mormidea collaris*				
Adult	90	–	10	31
*Mormidea* aff. *ypsilon*				
Adult	89	–	11	28
*Euschistus* sp.				
Adult	84	–	16	44
*Arvelius porrectispinus*				
Adult	78	–	22^d^	18
*Loxa* sp.				
Adult	86	–	14	44
**EDESSINAE**				
*Edessa* n. sp. 1				
Adult female	–	100	–	40
Adult male	–	100	–	43
Nymph (5th instar)	–	97	3	29
Nymph (3rd instar)	–	100	–	27
Egg	–	98	2	48
*Edessa bugabensis*				
Adult	–	100	–	27
Adult	–	100	–	26
*Edessa jugata*				
Adult	–	100	–	31
*Edessa* aff*. irrorata*				
Adult	–	100	–	27
Adult	–	100	–	29
*Edessa junix*				
Adult	–	97	3	28
*Edessa eburatula*				
Adult	–	100	–	24
**DISCOCEPHALINAE**				
*Antiteuchus costaricensis*				
Adult	–	100	–	20
Adult	–	100	–	19

a This is a novel lineage within the Enterobacteriaceae, most related to other Heteropteran symbionts.

b Pseudomonadales and Lactobacillales.

c Burkholderiales.

d Spiroplasma (Mollicutes).

### Phylogenetic analyses

Closest relatives were acquired using the GenBank Basic Local Alignment Search Tool, BLASTn (Altschul et al., 1997) and identified using phylogenetic analysis. Our 16S rRNA sequences along with additional sequences obtained from GenBank were compiled in ARB, after initial alignment using the SILVA Aligner function, with subsequent manual refinements (Ludwig et al., 2004; Preusse et al., 2007). For near full-length representatives and closest relatives, maximum parsimony (MP) analysis was conducted. In some cases, partial sequences recovered in our study were added to the tree in ARB via parsimony insertion within a tree of longer sequences. NJ analysis was also performed with 2000 bootstrap replicates to assign confidence levels to nodes, shown in Figure 2 (PAUP4.0b10; Swofford, 1998). Bacterial 16S rRNA sequences obtained in this study have been deposited in the GenBank database under accession numbers KJ934755-KJ934778.

### Transmission electron microscopy

For examination by transmission (TEM) electron microscopy, samples (~1 mm^3^) were fixed in 3% glutaraldehyde buffered with 0.2 M phosphate (pH 7.2). Following a wash in 0.1 M sodium cacodylate containing 24% sucrose, samples were post-fixed with 1% OsO_4_ in 0.1 M sodium cacodylate for 1 h, stained *en bloc* in 3% uranyl acetate in 0.1 M sodium acetate buffer for 1 h, dehydrated through an ethanol series, then infiltrated and embedded in Spurr's resin (Ted Pella, Redding, CA, US). Thick (0.4 μm) and thin (70 nm) sections were stained with methylene blue and lead citrate, respectively, then examined and photographed using a Zeiss Labrolux 12 light microscope and Zeiss EM109 TEM.

### Fluorescence *in situ* hybridization (FISH) microscopy

**Figure 3:**
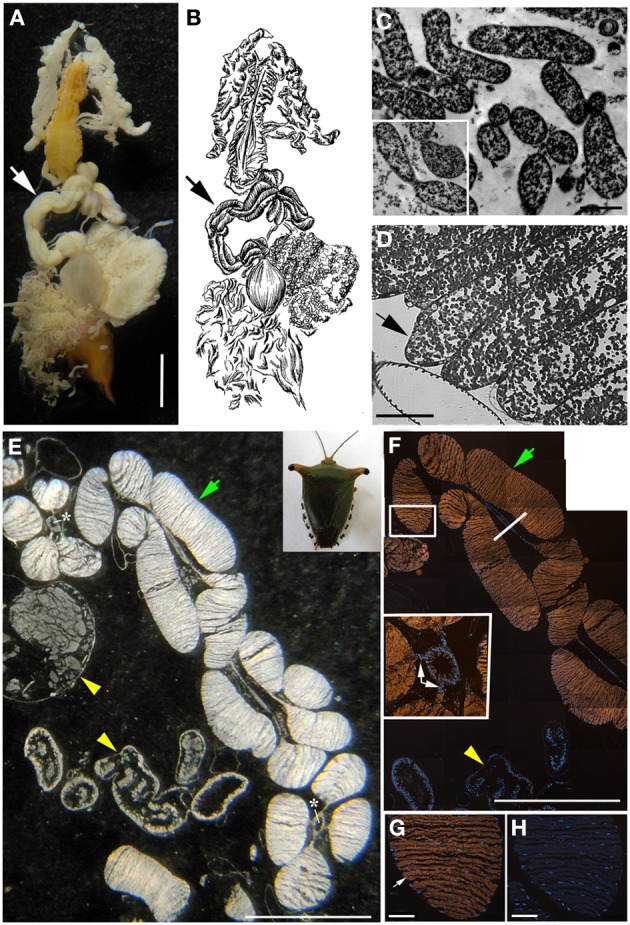
**Bacteria associated with *Edessa* n. sp. 1 (A)** Dissected digestive system, arrows indicate symbiont-bearing tissue. Scale, 2 mm **(B)** Line drawing of the digestive system **(C)** Transmission electron microscopy of rod-shaped bacteria-like cells within the midgut ceca. Scale, 2 μm. Inset shows bacteria dividing **(D)** Light microscopy showing bacteria-containing crypts within the midgut ceca (arrow). Scale, 20 μm. **(E)** A longitudinal section through the midgut ceca. The arrow denotes symbiont-containing midgut crypts, and the arrowheads are provided for reference to the non-symbiont containing tissues, including the upper left sac-like midgut region (M3) and the lower region of the hindgut, as denoted in Futahashi et al. (2013). Asterisks show the main gut cavity. Scale, 2 mm **(F)** Fluorescence *in situ* hybridization (FISH) microscopy of the same region as shown in E, hybridized with the Gam42 probe (Manz et al., 1992) labeled with Cy3, shown in orange, and counter-stained with DAPI, shown in blue. The square denotes the region shown in G/H. Scale, 2 mm. The line shows the location of the tissue in the inset in cross section. Inset shows connections between main gut cavity and crypts (arrows). **(G)** FISH microscopy of the region denoted in F, using the *Edessa*-symbiont specific probe labeled with Cy3, shown in orange, and counter-stained with DAPI, shown in blue. Scale, 100 μm. **(H)** FISH microscopy of the region denoted in F, as a negative control using an Epsilonproteobacteria-specific probe (EP404, Macalady et al., 2006), labeled with Cy3, shown in orange, and counter-stained with DAPI, shown in blue. Scale, 100 μm.

**Figure 4:**
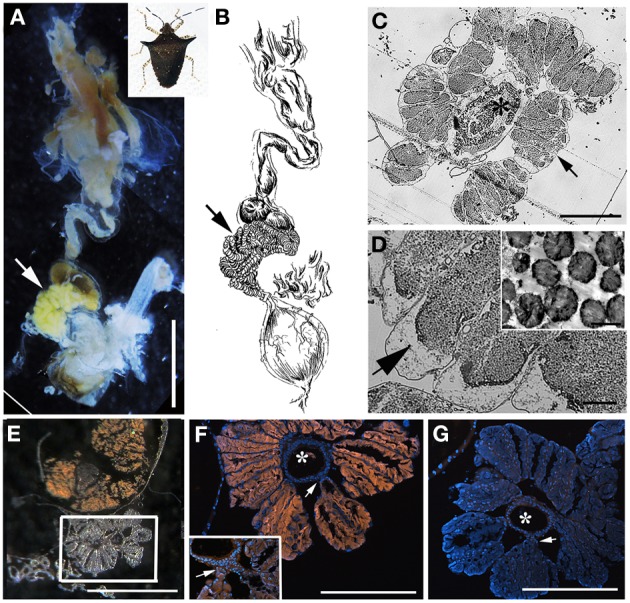
**Bacteria associated with *Sibaria englemani*. (A)** Dissected digestive system, arrows indicate symbiont-bearing tissue. Scale, 2 mm **(B)** Line drawing of the digestive system **(C)** Light microscopy image of a transverse section showing bacteria-containing crypts within the midgut ceca (arrow). An arrow indicates symbiont-bearing tissue and an asterisk shows the main gut cavity. Scale, 100 μm. **(D)** Transmission electron microscopy of bacteria-like cells within the midgut ceca. Scale, 20 μm. Symbiont separation from the outer tissue membrane, as denoted by the arrow, may be an artifact of fixation. Inset shows bacteria as irregular cocci (Scale, 2 μm). **(E)** A transverse section through the midgut ceca. The non-symbiont containing sac-like midgut region (M3), as described in Futahashi et al. (2013), is shown at top. The square denotes the region shown in **(F,G)**. Scale, 2 mm **(F)** Fluorescence *in situ* hybridization (FISH) microscopy of the same region as shown in **(E)**, hybridized with the *Sibaria*-symbiont specific probe labeled with Cy3, shown in orange, and counter-stained with DAPI, shown in blue. An asterisk shows the main gut cavity. Scale, 500 μm. Inset shows connections between main gut cavity and crypts (arrow). **(G)** FISH microscopy of the region shown in **(E,F)**, as a negative control using an Epsilonproteobacteria-specific probe (EP404, Macalady et al., 2006), labeled with Cy3, shown in orange, and counter-stained with DAPI, shown in blue. Scale, 500 μm.

**Figure 5:**
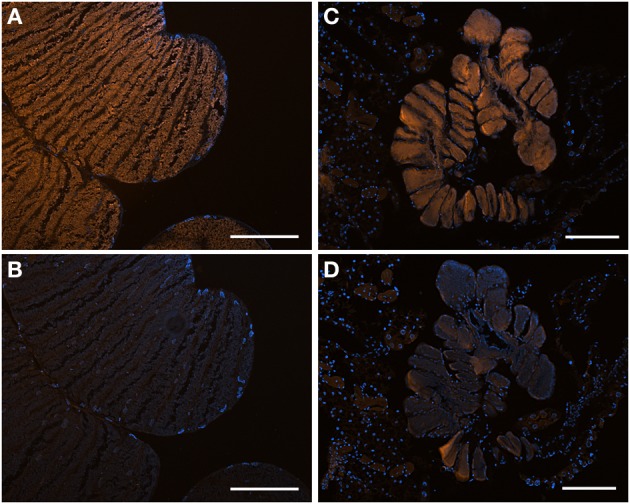
**Fluorescent *in situ* hybridization (FISH) images demonstrating specificity of the newly-designed probes. (A)** FISH microscopy of a section through the midgut ceca of *Edessa* n sp. 1, embedded in, and then removed from, Steedman's wax prior to hybridization, with the *Edessa*-specific probe labeled with Cy3, shown in orange, and counter-stained with DAPI, shown in blue. Signal intensity was maximum at 35% formamide, and was tested between 15–55% formamide. **(B)** The same section of *Edessa* n. sp. 1 midgut ceca, hybridized with the *Sibaria*-symbiont specific probe, labeled with Cy3, shown in orange, and counter-stained with DAPI, shown in blue. Note a lack of positive fluorescent signal. **(C)** FISH microscopy of a section through the midgut ceca of *Sibaria englemani*, embedded in, and then removed from, Steedman's wax prior to hybridization, with the *Sibaria*-specific probe labeled with Cy3, shown in orange, and counter-stained with DAPI, shown in blue. Signal intensity was maximum at 25% formamide, and was tested between 15–55% formamide. **(D)** The same section of *S. englemani* midgut ceca, hybridized with the *Edessa*-symbiont specific probe, labeled with Cy3, shown in orange, and counter-stained with DAPI, shown in blue. Note a lack of positive fluorescent signal. All scales = 100 μm.

For fluorescence *in situ* hybridization (FISH), insects, initially preserved in 4°C 4% paraformaldehyde for 24 h, were rinsed twice with 1X phosphate-buffered saline (PBS), transferred to 70% ethanol and stored at −20°C. Adult cecal tissue, or whole nymphs, were embedded in Steedman's wax (one part cetyl alcohol was added to nine parts polyethylene glycol (400) distearate, mixed at 60°C; Steedman, 1957), and added to the sample in an ethanol:resin gradient of 3:1, 2:1, and 1:1, according to Pernthaler and Pernthaler (2005). Samples eventually embedded in full strength wax were allowed to solidify and were sectioned (5–10 mm thick) using a Leica RM2125 manual microtome, and placed onto Superfrost Plus slides (Fisher Scientific). Samples were de-waxed by three rinses in 100% ethanol (5 min each), followed by rehydration in 70% ethanol (5 min). Hybridization and wash buffers were made as described previously (Pernthaler and Pernthaler, 2005), using 35% formamide in the hybridization buffer and 450 mM NaCl in the wash solution. A universal bacterial probe set was used (EUB338I-III, Amann et al., 1990; Daims et al., 1999), along with symbiont-specific probes targeting the 16S rRNA gene of the *Sibaria englemani* symbiont (Pentatominae-symbiont clade, Tribe Carpocorini, Figure 2; Sib_A, 5′-CCTG-GGCAGTTTC-3′), and the *Edessa* specific clade (Edessinae-symbiont clade, Figure 2; Ed_A, 5′-CCCTCCACTAGGTAGATCC-3′). The Sib_A probe had 100% identical sequence match to related *Erwinia/Pantoea*-like bacteria in the GenBank database, and the Ed_A probe exhibited only one mismatch to other Heteropteran symbionts, with no other obvious hits. Both were designed to have a Tm of ~53°C, GC = 58–61%. Formamide stringency was determined empirically by testing each probe against its target, and reciprocally against its non-target, between 15–55% formamide. Signal of symbiont-specific probes was strongest at 35 and 25% formamide, for the Ed_A (Figure 3) and Sib_A (Figure 4) probes, respectively, without compromising stringency (Figure 5). A probe targeting Epsilonproteobacteria (EP404_AAAKGYGTCATCCTCCA; Macalady et al., 2006) was used as a negative control. All probes were labeled with Cy3. Hybridizations were conducted at 46°C for 2–8 h, followed by a 15 min wash at 48°C. Tissues were counter-stained with a dilute 4′6′-diamidino-2-phenylindole (DAPI) solution (5 mg ml^−1^) for 1 min and examined under epifluorescence microscopy using a Nikon E80i epifluorescence research microscope with a Nikon DS-Qi1Mc high sensitivity monochrome digital camera.

### Host insect mitochondrial cytochrome c oxidase subunit I sequences

In order to confirm the identity of egg masses and nymphs collected in the field, insect mitochondrial cytochrome c oxidase subunit I (COI) was amplified using one of two previously published sets of primers; insectCOIF (5′-TACAATTTATCGCCTAAACTTCAGCC-3′) and insectCOIR (5′-CCCGGT-AAAATTAAAATATAAACTTC-3′; Kuechler et al., 2011) or LCO1490 (5′-GGTCAACAAA-TCATAAAGATATTGG-3′) and HCO2198 (5′-TAAACTTCAGGGTGACCAAAAAATCA-3′; Folmer et al., 1994). Thermal cycling conditions included 60 s each of denaturation at 94°C, annealing at 50 and 48°C, respectively, elongation at 72°C (25 cycles), and a final extension at 72°C for 6 min. COI gene products were cleaned prior to direct sequencing with MultiScreen HTS plates (Millipore Corporation, Bedford, MA). Samples were sequenced via ABI sequencing technology (Laragen, Inc., Culver City, CA). Sequences were assembled, edited, and aligned using Sequencher v4.10.1 (GeneCodes Corp.). For *Edessa* n. sp. 1, all individuals, including eggs, nymphs, and adults, displayed less than 0.3% divergence in their COI sequences (3 out of 850 bp), confirming that they all belonged to the same species. Similarly, all specimens thought to be *Sibaria englemani*, including eggs, nymphs, and adults, had less than 0.4% divergence in their COI sequences (3 out of 730 bp). Insect cytochrome c oxidase (COI) sequences obtained in this study have been deposited in the GenBank database under accession numbers KM020416-KM020425.

### Captive rearing and early development of costa rican pentatomids

**Figure 6:**
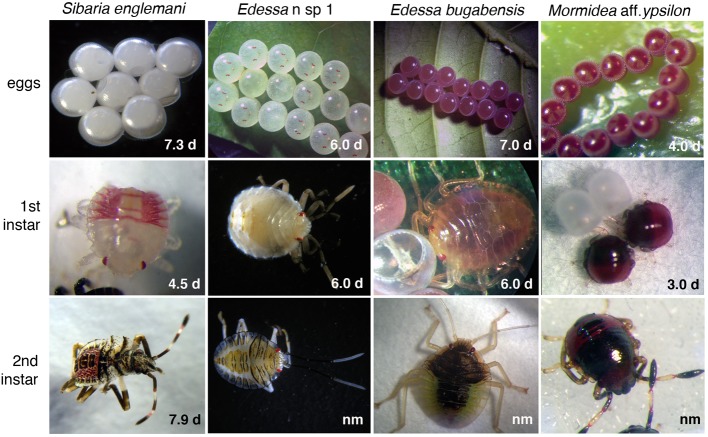
**Developmental series for four species used in this study**. Developmental duration for each life stage is noted. Average egg diameter, 1st instar length, and 2nd instar length was as follows: *Sibaria englemani* = 1.0, 1.3, 2.0 mm; *Edessa* n sp. 1 = 2.5, 3.0, 4.1 mm; *Edessa bugabensis* = 2.4, 3.1, 4.4 mm; *Mormidea* aff. *ypsilon* = 0.6, 0.7, 1.0 mm, respectively. nm, Developmental duration for 2nd instars of all species other than *S. englemani* are unknown, due to high mortality at this stage and unsuccessful metamorphosis to the 3rd instar.

Field-collected *Sibaria englemani* were housed in 14 × 14 × 24″ mesh enclosures under ambient conditions (24–32°C, 68–85% humidity, 12:12 h photoperiod) and supplied, every other day, with inflorescences of their native host plant, *Piper sancti-felicis*. Egg masses were removed from the enclosures within 12 h of oviposition and separated into one of several treatments to evaluate the mechanism of symbiont acquisition and impacts of symbiont deprivation (described below). Eggs and first instar nymphs (*n* = 58 egg masses from 190 females over 3.5 months) were incubated at 25–30°C in 3.5 cm petri dishes lined with filter paper, with wetted cotton for humidity. Second instar nymphs were then reared under the ambient conditions noted above. Populations of *Mormidea* aff. *ypsilon, Edessa* n. sp. 1, and *Edessa bugabensis* were also maintained on their respective host plants, to observe breeding and developmental progression of the nymphs (Figure 6).

### *Sibaria englemani* symbiont acquisition

Within ~6 h of hatching, *Sibaria englemani* cohorts (i.e., eggs from the same clutch; *n* = 4) were divided, with half of the 1st instar nymphs transferred to an identical petri dish to prohibit symbiont acquisition from the surface of the egg mass (chorion). First instar nymphs of the same cohort that were provided or denied access to the chorion were preserved ~24 h after hatching in 70% EtOH. Diagnostic PCR with symbiont-specific probes was conducted for both treatments to determine whether chorion probing facilitated symbiont acquisition.

### *Sibaria englemani* symbiont deprivation

To evaluate the impacts of symbiont deprivation on host development, *Sibaria englemani* eggs were surface sterilized upon hatching to prevent symbiont uptake by half of each cohort (*n* = 54 cohorts). Surface sterilization involved 5 min immersion in 10% bleach, modified from Prado et al. (2006). Symbiotic (control) eggs of each cohort were washed in sterile dH_2_O. Previous studies suggest that washes of the chorion are not directly harmful to the insect itself (Prado et al., 2006; Prado and Almeida, 2009b). Upon hatching, all first instar nymphs were allowed to probe the chorion to attempt symbiont acquisition. Second instar nymphs were relocated to independent petri dishes (3.5 cm) and nymphs assigned the symbiont-deprivation treatment were provided with antibiotic-laden water (0.005% kanamycin/ampicillin; Kuriwada et al., 2010). Congruously reared symbiotic and symbiont-deprived siblings were compared within cohorts to determine the effects of symbiont knockdown.

### Statistical comparisons of symbiotic and symbiont-deprived *sibaria englemani* nymphs

**Figure 7:**
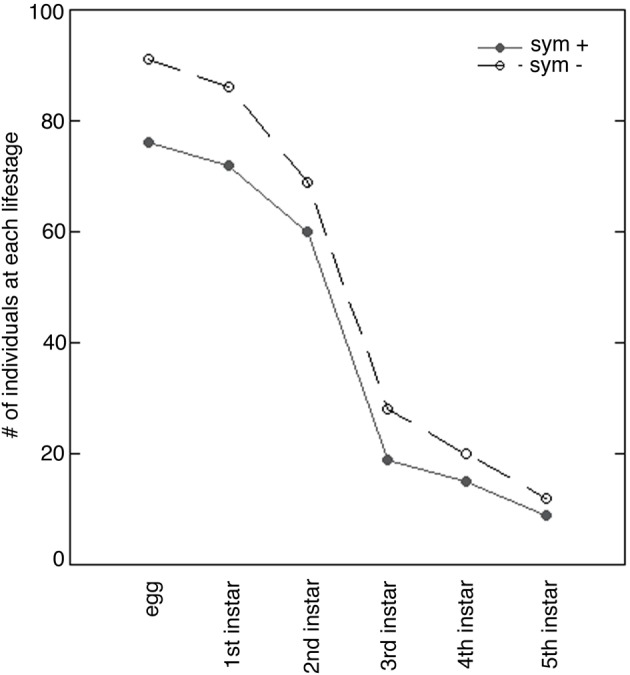
**Survivorship of symbiont-endowed (sym +) and symbiont-deprived (sym −) *Sibaria englemani* nymphs**. Number of nymphs per instar from emergence through the 5th instar indicates high mortality at the 2nd stadium when reared in captivity. Parallel survivorship curves represent no significant difference between symbiont-endowed (filled circles) and symbiont-deprived populations (unfilled circles; *p* = 0.96, Pearson's chi-squared test).

Developmental duration, morphometrics, and survivorship were compared between symbiotic and symbiont-deprived *Sibaria englemani* nymphs to determine if bacterial symbionts play a role in nymph development. Stadia duration was evaluated using a series of nonparametric two-sample tests (Wilcoxon's rank tests) with a Bonferroni correction (*p* = 0.01 significance threshold) for each instar, due to the non-normal distribution of stadia duration data. Survivorship was compared using a contingency table with no fixed margins, and analyzed via a Pearson's chi- squared goodness of fit test (Figure 7). Size of the insect was defined as the product of pronotum width and body length (mm). This morphometric was plotted against the age of the insect (time from hatching), to obtain average growth curves for both symbiotic and symbiont-deprived nymph populations. The slopes of linearized (log-transformed) growth curves for each individual were compared for all nymphs that reached the third instar (*n* = 39), and evaluated using a two-sample *t*-test: data fulfilled assumptions of normality and variance for each treatment. Comparative analyses were performed using the Java Graphical User Interface for R (JGR v.1.7-16; GNU General Public License). Phylogenetic congruence between host and symbiont phylogeny was evaluated using the Icong index of topological similarity (de Vienne et al., 2007).

## Results

### Bacteria associated with costa rica pentatomid stinkbugs

**Figure 8:**
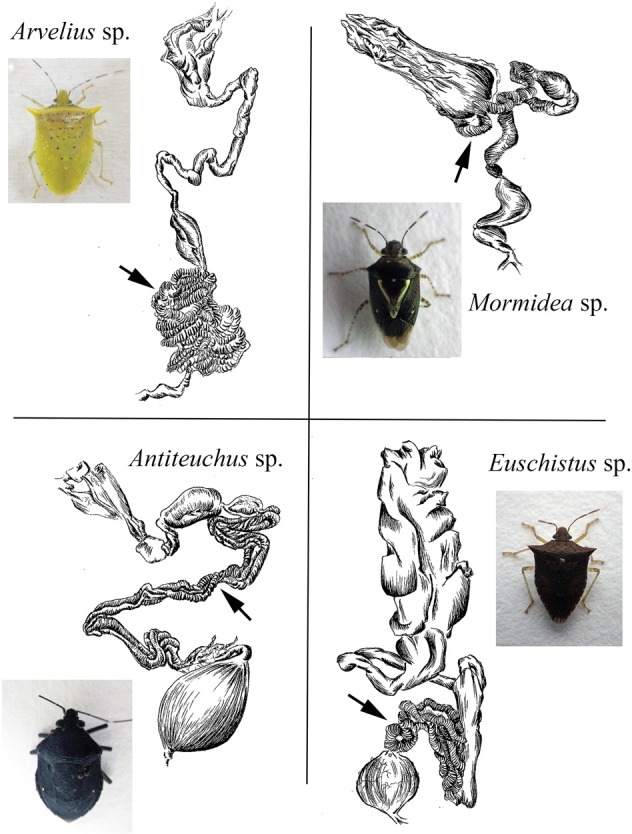
**Line drawings of the digestive tract of four species examined in this study**. Arrows denote the symbiont-containing ceca, with crypts. Similar sketches for *Sibaria englemani* and *Edessa* n sp 1 are shown in Figures 3, 4.

The bacteria-bearing organ, or ceca, was conspicuous in all specimens, with numerous tube- or sac-like convolutions (crypts) arranged in a coiled rosette (Figures 3, 4, 8). The ceca comprised 8–20% of the soft tissue biomass of *Edessa* specimens, whereas it comprised ~5% in *Sibaria* specimens. Crypts, which varied in size, color, and configuration between species, housed extracellular symbionts in the lumen (Figures 3, 4, 8). The cecal organ was morphologically distinct between host genera but not between closely related taxa within subfamilies. For example, crypts of the subfamily Edessinae were pale yellow in color with large and neatly aligned in two fused rows with few convolutions, in comparison to the translucent and elaborately folded crypt cavities of the subfamily Pentatominae (Figures 3, 4, 11). To determine the identity of microbes associated with each stinkbug species, 16S ribosomal RNA sequences were amplified from ceca DNA extracts, as well as extracts from whole eggs and nymphs (Figure 2). Results show that all Costa Rican pentatomids possessed a dominant gammaproteobacteria. Gut bacteria were polyphyletic with three primary clades generally congruous within subfamily designations of the stinkbug hosts. The Icong congruence index for testing topological similarity between trees revealed a *P*-value of 0.06 (de Vienne et al., 2007). For example, bacteria closely related to the cultured genus *Erwinia* were dominant within the subfamily Pentatominae, based on 98% similarity, including *Sibaria englemani* (adults, nymphs, and oviposited eggs), *Mormidea collaris, M.* aff. *ypsilon, Euschistus* sp., *Arvelius porrectispinus, and Loxa* sp., representing 78–98% of the recovered 16S rRNA sequences associated with each specimen (Figure 2, Table 1). Bacteria associated with these six stinkbug species were >97% similar to each other in 16S rRNA sequence. Further, similarity among bacterial associates was observed for the tribe Carpocorini, subfamily Pentatominae (only 1% divergence in 16S rRNA sequence; Figure 2), despite the very different diets among hosts, including grasses within the Poacea for *Mormidea* and *Euschistus* and *Piper sancti-felicis* for *Sibaria englemani.* The presence of a unique, dominant bacterial type within the Enterobacteriaceae (97–100% of recovered 16S rRNA sequences; Table 1), monophyletic and equidistant (based on ~90–94% similarity) between symbionts of the plataspid stinkbug *Megacopta punctatissima* (*Candidatus* Ishikawaella capsulata; Fukatsu and Hosokawa, 2002) and the brown marmorated stinkbug *Halyomorpha halys*, was also confirmed for six species within the subfamily Edessinae and *Antiteuchus costaricensis*, within the subfamily Discocephalinae (*n* = 1–4 individuals of each species; Figure 2, Table 1). Bacteria associated with the six *Edessa* species were >93% similar to each other in 16S rRNA sequence, while the ribotype associated with *Antiteuchus* was slightly less similar (91–93% of the 16S rRNA sequence). Overall, the two main bacterial clades associated with *Sibaria* and *Edessa* shared only ~88–92% similarity, based on 16S rRNA sequence.

**Figure 9:**
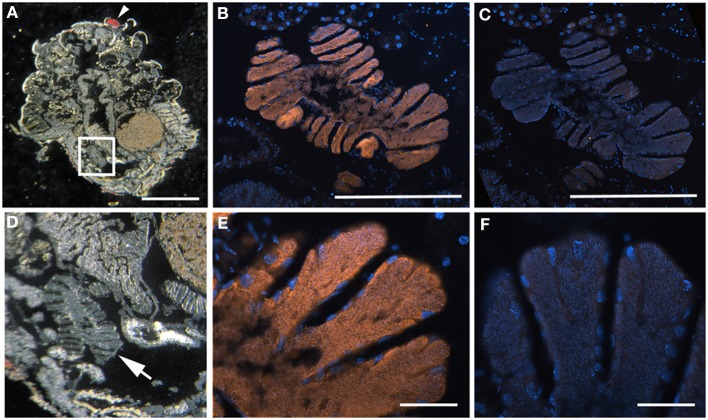
***Sibaria englemani* nymph FISH microscopy. (A)** Light images of section through an entire *S. englemani* nymph, embedded in, and then removed from, Steedman's wax prior to hybridization. The anterior of the specimen (eye) is denoted by the arrowhead. The box denotes the region shown in **(B,C)**. Scale, 1 mm. **(B)** Fluorescence *in situ* hybridization (FISH) microscopy of the same region as shown in E, hybridized with the *Sibaria*-symbiont specific probe labeled with Cy3, shown in orange, and counter-stained with DAPI, shown in blue. Scale, 100 μm. **(C)** FISH microscopy, as a negative control using an Epsilonproteobacteria-specific probe (EP404, Macalady et al., 2006), labeled with Cy3, shown in orange, and counter-stained with DAPI, shown in blue. Scale, 100 μm. **(D–F)** Magnified versions of **(A–C)**, respectively. Scales are 10 μm for **(E,F)**.

**Figure 10:**
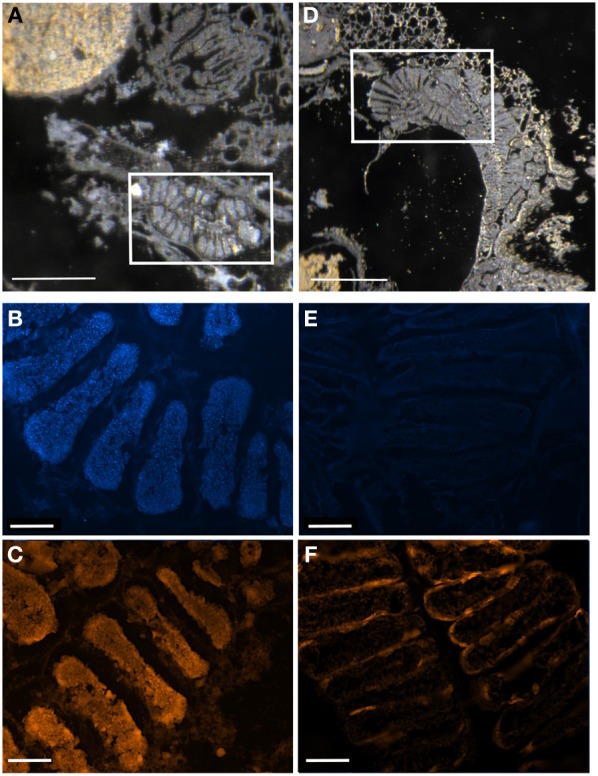
**Digestive ceca of symbiotic vs. symbiont-deprived 5th instar *Sibaria englemani* nymphs. (A–C)** Light microscopy, DAPI, and FISH microscopy, respectively, of the ceca of a symbiotic nymph, showing dense bacteria within the crypt spaces. **(A)** shows the non-symbiont containing sac-like midgut region (M3), as described in Futahashi et al. (2013), at top left **(D–F)** Light microscopy, DAPI, and FISH microscopy, respectively, of the ceca of a symbiont-deprived nymph, showing lack of cecal bacteria and visual differences in gross ultrastructure. **(A,D)** Light images of sections through the midgut ceca, embedded in, and then removed from, Steedman's wax prior to hybridization. Boxes denote areas of subsequent DAPI and fluorescent 16S rRNA probing. **(B,E)** Fluorescent images showing boxed regions in **(A,D)** counter-stained with DAPI, observed in blue. **(C,F)** Fluorescence *in situ* hybridization (FISH) microscopy of the boxed regions in **(A,D)**, hybridized with the Eub338 probe set labeled with Cy3, shown in orange. Light image scales, 2 mm. Fluorescent image scales, 20 μm.

For both *Sibaria englemani* and *Edessa* n sp. 1, the dominant bacterium was detected in all lifestages, including oviposited eggs and nymphs hatched in environmental isolation in the laboratory (Table 1). Dissection microscopy and FISH was utilized to locate and visualize symbionts in *Sibaria englemani* and *Edessa* n sp. 1. Transmission electron microscopy revealed the presence of bacteria-like cells of a single morphotype, within the ceca of both species (Figures 3, 4). The midgut symbionts within *Edessa* n sp. 1 were rod shaped, approx. 1.0–4.5 μm in length, and filled the entire lumen within each crypt (Figures 3C,D). The midgut symbionts within *S. englemani* were cocci shaped, approx. 1.0–1.5 μm in diameter (Figures 4C,D). Similarly, FISH microscopy with a specific probe revealed that a dominant gammaproteobacteria populated the lumen of crypts in both insects (Figures 3, 4). Bacterial colonization of specific midgut regions provided compelling evidence of a genuine symbiotic relationship. Fluorescence microscopy also revealed crypt-containing, symbiont-filled tissues, albeit much smaller than in adults, in a wild caught *S. englemani* 4th instar (Figure 9) and in 5th instar nymphs reared in the laboratory (Figures 10A–C). The ceca comprised ~1–2% of soft tissue biomass of *Sibaria* nymphs, as compared to ~5% in adults. Connections between the midgut main cavity and the symbiont-filled crypts were noted for both *Sibaria* and *Edessa* (Figures 3F, 4F), suggesting that the insects are able to excrete the symbiotic bacteria onto their eggs by surface contamination (Goodchild, 1963; Kikuchi et al., 2009).

### *Sibaria englemani* captive rearing and symbiont acquisition

**Figure 11:**
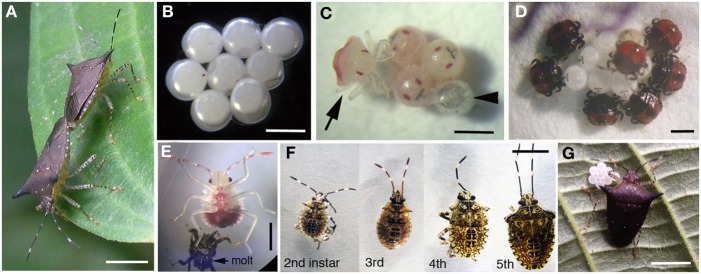
**Captive rearing of *Sibaria englemani*. (A)** Mating adults with female at bottom left. Scale, 5 mm **(B)** Eggs oviposited in mesh cages. Scale, 1 mm **(C)** Newly hatched nymph (arrow), with empty egg at arrowhead. Scale, 1 mm **(D)** Newly hatched nymphs probing outer egg surfaces. Scale, 1 mm **(E)** 2nd instar, post molting. Scale, 1 mm **(F)** 2nd–5th instars of *Sibaria englemani*. Scale, 5 mm **(G)** Female, with recently oviposited egg mass. Scale, 5 mm.

Adult *Sibaria englemani* collected in June 2013 were maintained in ambient conditions, at 25–30°C, for ~55 days and reproduced successfully in captivity when provided with inflorescences of their native diet, *Piper sancti-felicis.* Females preferentially oviposited on mesh cage surfaces, typically producing 8 eggs per cohort (~1 mm in diameter; Figures 6, 11), with an incubation time of 7.3 ± 0.2 d and ~95% viability. Egg masses were turgid and shiny, adhering to leaf surfaces or mesh caging. Eggs developed visible eyespots and ruptor ovis approximately 2–3 days prior to emergence. Stadia durations averaged 4.5 ± 0.6, 7.9 ± 2.9, 7.3 ± 1.7, and 7.5 ± 1.4 d (mean ± standard error) for 1st through 4th instars, respectively. Upon hatching, teneral first instar nymphs aggregated on the eggs (Figures 11C,D), and were observed to probe the chorion for 1–6 h after hatching (Supplemental Video). Diagnostic PCR amplification using symbiont-specific primers demonstrated that intact eggs contained symbionts, whereas neither the ovaries of females nor eggs with the chorion removed did (data not shown). Further, diagnostic PCR amplification of the symbiont 16S rRNA gene indicated that only nymphs allowed to probe the chorion successfully acquired symbionts, whereas those that were removed from their egg masses within 10 min of hatching did not, supporting maternal transmission as the predominant mechanism of symbiont acquisition.

### Impacts of symbiont deprivation on *sibaria englemani*

Complete symbiont-deprivation was not attained in all insects: ~50% of nymphs with surface sterilized chorions exhibited incomplete aposymbiosis (as revealed by PCR and QPCR amplification of symbiont specific 16S rRNA, data not shown). Several surface sterilization protocols, including immersion in 95% ethanol or 4% formalin prior to a wash in bleach also resulted in incomplete symbiont knockdown, despite reports of previous success in the literature (Bistolas, personal observation; Prado et al., 2006, 2009; Hosokawa et al., 2007). Overall, the process of surface sterilization of the chorion to knockdown symbionts did not impact total egg viability (95%). Survivorship of symbiont-deprived and symbiont-endowed nymphs was comparable (80 and 83%, respectively, *p* = 0.959, Pearson's Chi-squared-test), with both treatments experiencing high mortality at the second stadium when reared in captivity (Figure 7). Vulnerability to high mortality and failure to molt to the 3rd instar may be due to nutritional deficits or susceptibility to fluctuations in ambient conditions, as reported by studies on the Southern green stinkbug, *Nezara viridula* (Hokyo and Kiritani, 1963; Prado et al., 2009). Symbiont depression did not influence total developmental duration (34.0 ± 0.8 d vs. 37.0 ± 1.5 d, for symbiotic vs. symbiont-deprived nymphs, respectively). Stadia durations were equal for 1st and 3rd–5th stadia in both symbiotic and symbiont-deprived nymphs (*p* = 0.87, *p* = 0.26, *p* = 0.17, and *p* = 0.26, for first, third, fourth, and fifth instars respectively, Wilcoxon's rank). Standard length and pronotum width were comparable between symbiotic and symboint-deprived nymphs. Similarities in morphometrics and percent survivorship may be a reflection of incomplete symbiont knockdown.

**Figure 12:**
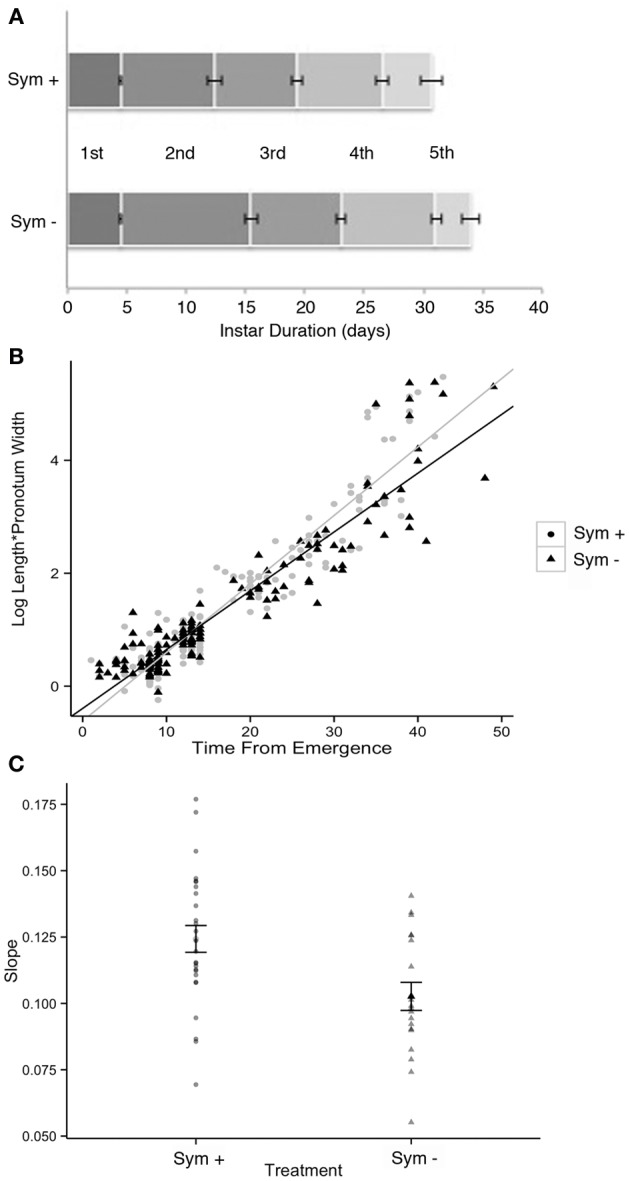
**Development of symbiotic (Sym +) vs. symbiont-deprived (via surface sterilization of the egg masses; Sym −) *Sibaria englemani* nymphs. (A)** Stadia durations were equal in both symbiotic and aposymbiotic nymphs for the first, third, fourth, and fifth instars (*p* = 0.87, *p* = 0.26, *p* = 0.17, and *p* = 0.26, respectively, Wilcoxon's rank). However, symbiont-deprived nymphs experienced significantly elongated second stadia (9.1 ± 2.9 d for aposymbiotic vs. 7.9 ± 2.9 d for symbiotic nymphs, *p* = 0.0001, Wilcoxon's rank with Bonferroni correction). Symbiont depression did not significantly influence total developmental duration (first to fifth instar). **(B)** Linearized growth rates for symbiotic and symbiont-deprived nymphs. **(C)** Averages of the slopes of the linearized growth rates from **(B)** (difference *p* = 0.005, Welch 2-sample *t*-test).

Despite incomplete aposymbiosis, congruent rearing of symbiont-endowed and symbiont-depressed siblings provided insight into effects of the symbiont on host development. Symbiont-deprived insects experienced significantly elongated second stadia (9.1 ± 2.9 d for symbiont-deprived vs. 7.9 ± 2.9 d for symbiont-endowed nymphs, *p* = 0.0001, Wilcoxon's rank with Bonferroni correction; Figure 12) and significantly slower linearized growth rates (*p* = 0.005, Welch 2-sample *t*-test), requiring more time to achieve larger sizes (standard length x pronotum width) compared to symbiotic siblings (Figures 12B,C). Further, surface-sterilized *S. englemani* nymphs exhibited symbiont-deprived ceca with observable differences in cecal morphology compared to control nymphs, using FISH microscopy (Figures 10D–F). In one case, a 1st instar nymph allowed to probe the surface of it's surface-sterilized egg, acquired *Burkholderia*, most closely related to soil-dwelling *B. cepacia* (KF974366), in replacement of the native symbiont, both of which were confirmed via 16S rRNA amplification and sequencing. *Burkholderia* was also observed to be among the bacteria associated with non-manipulated egg surfaces (7% of the community; Table 1; data not shown). Thus, while results assist in the resolution of the status of the symbiosis, more vigorous surface sterilization protocols may inform future observations of the impacts of symbiont deprivation on nymph ontogeny and morphometry.

## Discussion

A microbial examination of thirteen Costa Rican pentatomid species, which are taxonomically distinct from more well-studied temperate relatives, revealed near-monocultures of gammaproteobacteria within the family Enterobacteriaceae present in the lumen of crypts in the distal midgut of these insects. Association of each pentatomid species with an abundant, albeit variable, gammaproteobacteria is in agreement with previous observations for other Pentatomomorpha within at least six families (Hosokawa et al., 2006, 2010, 2012; Kikuchi et al., 2009; Kaiwa et al., 2010). The dominant bacterium associated with Costa Rican pentatomids was generally congruent with insect phylogeny (at the level of subfamily and tribes). Bacteria closely-related to *Erwinia* were associated with six species from the subfamily Pentatominae, including the genera *Sibaria, Mormidea, Euschistus, Arvelius, and Loxa*. These species feed mainly on fruits and seeds of herbs or small shrubs belonging to a few families (i.e., Fabaceae, Piperaceae, Poaceae, Solanaceae). Further clustering of gut bacteria within this subfamily was observed in one insect tribe, with a very closely-related group of bacteria associating uniquely with members of the Carpocorini (subfamily Pentatominae), despite diverse diets of the insect hosts (e.g., Poaceae vs. Piperaceae). On the other hand, a unique group of bacteria within the Enterobacteriaceae were associated with six species within the subfamily Edessinae, which are known to feed on phloem extracted from branches and leaves of trees and shrubs within the Mirtaceae, Sterculiaceae, Fabaceae, and Annonaceae.

Fluorescent and transmission electron microscopy confirmed the location of the midgut crypts and provided further insight into the differences between the cocci-shaped symbionts within *Sibaria englemani* (Figures 4C,D) and the rod-shaped symbionts within *Edessa* n sp. 1 (Figures 3C,D). Although not visualized, an additional bacterial type, closely related to the Edessinae symbionts, was observed for the single species of Discocephalinae, *Antiteuchus costaricensis*. Unlike the explored *Edessa* and *Sibaria* species, *Antiteuchus* species are able to colonize plants from several different families (primarily Malvaceae, but sometimes Sterculiaceae, Combretaceae, Mirtaceae, Euphorbiacea) always piercing branches, leaves and growing fruits.

Symbiont identity aligned with host phylogenies to the level of insect subfamily, and tribe. Inclusion of symbionts from additional pentatomid tribes collected in Japan and Europe (including Nezarini, Strachiini, and Cappaeini; Figure 2) however, added a level of complexity that obscured the congruence at the family level. As in previous studies, bacteria found in association with other insects, including Thripidae, Psyllidae, Cydnidae, and Scutteleridae also grouped within the Pentatomidae bacterial clade, further complicating our understanding of the role of insect phylogeny on shaping the gut microbial community. The extraembryonic egg casing, or chorion, functions as a conduit for vertical symbiont acquisition in several pentatomids, including *Nezara viridula* (Prado et al., 2006; Tada et al., 2011), Eurydema spp. (Kikuchi et al., 2012), *Acrosternum hilare* (Buchner, 1965; Prado et al., 2006; Prado and Almeida, 2009b), and *Plautia stali* (Abe et al., 1995). Maternal transmission through smearing of the chorion with symbionts after oviposition was apparent in *S. englemani*, as observed previously in other pentatomids, supported by symbiont presence in all life stages and in the chorion (but not embryonic or ovarian tissues; Prado et al., 2006; Kikuchi et al., 2012; Prado and Zucchi, 2012). The egg smearing strategy, however, clearly affords the possibility of bacterial “contamination” from the environment and possible symbiont replacement over evolutionary timescales. Connections between the midgut central cavity and the crypts were observed in both *Sibaria* and *Edessa*, and must allow not only symbiont deposition onto the eggs during oviposition, but also the occasional colonization of non-symbiotic bacteria acquired transiently via foraging. Maintenance and persistence of a novel symbiont is likely to only occur with other taxonomically similar bacteria. However, exposure to many types of bacteria during feeding and roaming is expected. This possibility is supported by our observation of a single occurrence of a soil bacterium, most closely related to *Burkholderia cepacia*, in the midgut of a *Sibaria englemani* nymph that, upon hatching, was only allowed to probe the surface of sterilized eggs (data not shown).

The variable nature of the primary bacterial symbionts between pentatomid species is similar to that observed for the nutritional symbionts of Cydnidae (burrower bugs), which have undergone substitution many times during their evolution (Hosokawa et al., 2012). Whether infection frequencies of the various symbionts correlate with internal (host physiology, diet) or external (climatic and ecological) influences largely remains unknown. For *Edessa* n sp. 1 found on both *Handroanthus* and *Pentaclethra*, identical midgut bacteria were observed, further suggesting that host identity is more influential than diet on gut bacterial composition, at least on short time scales. The latter perhaps falsely presumes that the host plant upon which the insects are collected is the source of the diet, however, these two populations had different phenotypes (antennae color) and were regularly (i.e., non-randomly) observed on the disparate host plants.

Phylogenetic studies have provided evidence of a continuum between plant pathogens and insect symbionts (reviewed in Frago et al., 2012). For example, members of the genus *Erwinia*, which comprise a distinct phylogenetic group, cause soft rot, necroses, and wilt on a variety of plants (Starr and Chaterrjee, 1972). Pectolysis might be an advantage to an herbivorous insect if, over evolutionary time, a specific association was formed with these particular types of bacteria. For incomplete vertical transmission (e.g., egg smearing and nymph ingestion), plant surfaces and interiors, for species that feed by piercing into the plant's vascular system, may act as passive sources of novel bacteria, whereby successful inoculation of juvenile gut flora could occur between closely related bacterial species. Whether these associations represent a new specialized role for the bacterium as an insect mutualist or a continuum of interactions between bacteria, insect and plant is not known. In the case of *Sibaria englemani*, bacteria very similar to the symbiont (within 99% similarity based on 16S rRNA) were present on an influorescence of *Piper sancti-felis*, the only known host plant of this particular insect species, yet this bacterial ribotype made up less than 1% of the community (data not shown). A comparison between insect symbionts and close relatives in the environment would provide the opportunity to explore their patterns of adaptation and ecological diversification.

Rearing of both symbiotic and symbiont-deprived siblings, established by egg surface sterilization, provided insight into the effects of symbiont deprivation on host development and survivorship. Despite incomplete surface sterilization of egg mass surfaces, *Sibaria englemani* nymphs deprived of most symbionts experienced significantly extended second stadia, resulting in delayed growth rates. Adverse effects of symbiont knockdown indicate that the microbial partner positively influences nymph ontogeny to some degree, although the details of their role remain unclear. Beneficial associations between these insects and bacteria include a wide range of specific interactions, from protective to nutritional, thereby providing ecological niches that would have otherwise been un-exploitable. For example, *S. englemani* preferentially utilizes the allelochemical-rich reproductive structures or nutritionally-deplete vascular fluids of tropical pipers (Whitehead and Bowers, 2014), and may benefit from diet detoxification or dietary supplementation by the symbiont. However, because symbiont knockdown did not impact the mortality of *S. englemani* reared on host plants rich in toxic secondary metabolites (*Piper sancti-felicis*; Bistolas, personal observation), it is unlikely that symbionts play a role in diet detoxification. In insects subsisting on unbalanced diets, or those of low digestibility or nutritional value, symbionts are known to provide the host with limiting nutrients, such as amino acids, vitamins, and useable nitrogen (Douglas, 1998; Wu et al., 2006; Pais et al., 2008; Russell et al., 2009). Since the synthesis of select metabolites by bacterial symbionts is particularly important for phloem- and xylem-feeding insects (Nakabachi and Ishikawa, 1999; Moran et al., 2003; Urban and Cryan, 2012), including the related plataspid stinkbug *Megacopta punctatissima* (Hosokawa et al., 2006), it is expected that pentatomid stinkbugs might also depend upon nutrient provisioning by gut bacteria.

## Conclusion

Near-monocultures of gammaproteobacteria form non-transient associations with seven genera of neotropical pentatomid hosts (*Antiteuchus, Arvelius, Edessa, Euschistus, Loxa, Mormidea*, and *Sibaria)*, colonizing the lumen of a modified midgut organ, or cecum. There is a correspondence between polyphyletic symbiont and host insect phylogenies among 13 micro-allopatric host species. Coupled with observations of a modified midgut organ, intermediate maternal symbiont transmission through the chorion, and negative impacts of symbiont knockdown on early-instar development in one species, *Sibaria englemani*, these results indicate that gut microbiota confer benefits to the host. The ubiquity and potential function of such symbioses in neotropical pentatomids suggest the occurrence of microbially-mediated diversification (“micro-allopatric” speciation through specialization; Fitzpatrick et al., 2008) by allowing insects to take advantage of underutilized or otherwise inaccessible resources, such as toxic or nutrient-deplete host plants. By influencing insect functional ecology and patterns of herbivory, such interdomain symbioses may play an integral role in the stability of neotropical ecosystems (Coupe and Cahill, 2003; García-Robledo et al., 2013; Metcalfe et al., 2014). Further exploration of both the ecological relationship (symbiont function) and coevolutionary history between insect and microbe may provide evidence that mutualistic symbionts facilitate host plant specialization and inform global patterns of arthropod biodiversity.

### Conflict of interest statement

The authors declare that the research was conducted in the absence of any commercial or financial relationships that could be construed as a potential conflict of interest.

## Supplementary material

Supplemental Video ***Sibaria englemani* symbiont acquisition**. Newly hatched (5–10 min post-emergence), teneral first instar nymph demonstrating chorion probing behavior. Nymph probing provides evidence of oral symbiont acquisition via vertical transmission of symbionts smeared on the chorion exterior.
